# Astrocytes and Microglia and Their Potential Link with Autism Spectrum Disorders

**DOI:** 10.3389/fncel.2016.00021

**Published:** 2016-02-12

**Authors:** Francesco Petrelli, Luca Pucci, Paola Bezzi

**Affiliations:** Department of Fundamental Neurosciences, University of Lausanne, Lausanne, Switzerland

**Keywords:** astrocytes, microglia, neurosciences, autism spectrum disorders (ASD)

## Abstract

The cellular mechanism(s) underlying autism spectrum disorders (ASDs) are not fully understood although it has been shown that various genetic and environmental factors contribute to their etiology. As increasing evidence indicates that astrocytes and microglial cells play a major role in synapse maturation and function, and there is evidence of deficits in glial cell functions in ASDs, one current hypothesis is that glial dysfunctions directly contribute to their pathophysiology. The aim of this review is to summarize microglia and astrocyte functions in synapse development and their contributions to ASDs.

## Introduction

Autism spectrum disorders (ASDs) have a worldwide prevalence of about 12-15% and are characterized by significant social, intellectual, behavioral impairment, and sometimes cognitive deficits (Abrahams and Geschwind, 2008; Geschwind, 2009; Quaak et al., 2013). They typically manifest early in development and are associated distinct neurodevelopmental syndromes.

The conceptualisation of autism and related disorders has undergone considerable changes over the last ten years, which are reflected in the fifth edition of the Diagnostic and Statistical Manual of Mental Disorders (DSM-V, www.dsm5.org). The proposed revisions of the preceding edition (DSM IV-TR) include combining specific DSM-IV-TR diagnoses into a single broad ASD, and identifying two domains of impairment (social communication and interactions, and restricted repetitive behavior) rather than three (social interaction, communication, and restricted repetitive and stereotyped patterns of behavior, interests, and activities). There are also considerable differences in clinical presentation and disease progression as ASD patient’s present variously severe core symptoms and variable co-morbid conditions such as epilepsy, gastrointestinal problems, intellectual disability, anxiety, and depression (Kim and Lord, 2012). Early estimates that the heritability of ASDs is approximately 90% (Steffenburg et al., 1989; Bailey et al., 1995), and even recently revised estimates that it is about 45%, strongly suggest that genetic mutations are a major cause (Hallmayer et al., 2011; Sandin et al., 2014). However, ASDs are genetically very heterogeneous (State and Levitt, 2011) and seem to be associated with a large number of genetic mutations, including possibly 100s of rare causal variants and common variants with small effects (Murdoch and State, 2013). The genetic heterogeneity of ASD has made it challenging to identify specific genes associated with the disorder, which has thus hindered efforts to dissect disease mechanisms. Recent insights into the genetic pathways that are altered in ASDs have come from studies of syndromic disorders with a high incidence of ASDs caused by mutations of a single gene, including fragile X syndrome (fragile X mental retardation 1 protein, FMR1), Rett syndrome (methyl-CpG-binding protein 2 protein, MECP2), tuberous sclerosis (tuberous sclerosis 1 protein, TSC1), neurofibromatosis type 1 (neurofibromin 1 protein, NF1), and PTEN (phosphatase and tensin homologue) macrocephaly. Recent studies also implicate neurodevelopmental genes in ASDs through the identification of recurrent *de novo* loss of function mutations in affected individuals (Parikshak et al., 2013; Willsey et al., 2013). Neurodevelopmental genes are indeed an important factor to take into consideration since functional and anatomical insults associated to defects in these genes during brain development can trigger the appearance of ASDs in the childhood. Genome-wide association studies have identified susceptibility genes for ASDs such as forkhead box p2 (FOXP2) (Toma et al., 2013) or the MAM domain containing glycosylphosphatidylinositol anchor (MDGA) genes (Bucan et al., 2009; Pettem et al., 2013; Perez-Garcia and O’Leary, 2016) and have provided evidence supporting the idea that the large numbers of variants associated with ASDs converge toward a core set of dysregulated biological processes (Murdoch and State, 2013). The genes that have been linked to ASDs can be grouped into three broad categories: those involved in synapse structure and activity (Etherton et al., 2011a,b; Peca and Feng, 2012), those involved in protein synthesis (Kelleher and Bear, 2008), and those involved in regulating gene expression (van Bokhoven, 2011). Many of them encode for proteins that have a clear synaptic function, thus making the pathological features of ASDs mainly “neurocentric”. However, as the usefulness of parsing neuronal mechanisms in order to investigate the etiology of ASDs has proved to be limited, alternative biological analyses may help to reveal previously unknown cellular and molecular mechanisms.

Extending the theory of purely genetic causes, it seems likely that genetics alone may not account for all cases of autism. In addition to a certain combination of autism-related genes, exposure to a number of non-heritable environmental factors may significantly affect susceptibility to, and the variable expression of autism and autism-related traits (Pessah and Lein, 2008; Rosenberg et al., 2009; Hallmayer et al., 2011; Estes and McAllister, 2015). In addition to exposure to chemicals or toxins, these include factors such as parental age at the time of conception, and maternal nutrition and infections (including autoimmune diseases) during pregnancy and prematurity (Grabrucker, 2012). One important area of research concerns the way in which environmental influences interact with genetic susceptibility: for example, recent studies have shown that glial cells in the brains of autistic subjects were constantly activated and their inflammation response genes were turned on (Voineagu et al., 2011; Edmonson et al., 2014; Gupta et al., 2014). It is still not clear what role inflammation plays in autism or whether it is beneficial or not, or what causes the activation of glial cells, but these findings and the recent discovery that glial cells may interact with synaptic activity by influencing synapse formation and maturation (Clarke and Barres, 2013; Sahlender et al., 2014; Petrelli and Bezzi, 2016), strongly suggest that glial cells may be involved in the pathogenesis of ASDs. Although some excellent reviews have recently discussed the involvement of glial cells in neuropsychiatric disorders (McGann et al., 2012; Molofsky et al., 2012; Chung et al., 2015), we will give our perspective on the role of microglia and astrocytes in the late step of synapse formation and maturation and in the pathophysiology of ASDs.

## Astrocytes And Microglia Influence Synapse Formation And Function

Over the last ten years, it has gradually emerged that glial cells (in particular microglia and astrocytes) influence synapse formation and function (Araque et al., 2014; Chung et al., 2015; Rossi, 2015). Neurons and glial cells are closely associated with each other from an early stage of development, and recent discoveries suggest that the appropriate assembly of neural circuits requires extensive neuron-glia signaling (Ullian et al., 2001; Christopherson et al., 2005; Eroglu, 2009; Allen et al., 2012). In addition to carrying out various homeostatic functions within the central nervous system (CNS), they can also engage in bi-directional communication with neurons by releasing neuroactive substances (Petrelli and Bezzi, 2016). In particular, astrocytes can make contact with multiple neurons and up to 100,000 synapses (Bushong et al., 2002; Halassa et al., 2007), and possess many receptors (mainly G-protein coupled receptors) and ion channels present in neurons, thus enabling them to sense and respond to an array of neuronal signals (Fiacco and McCarthy, 2006). The generation and expansion of astrocytes is largely completed before birth, but the elaboration and maturation of their fine peri-synaptic processes persists during the active period of synaptogenesis (Ullian et al., 2001) in the post-natal period. This suggests that they are in a crucial position to communicate actively with neurons during synaptogenesis and, thus, coordinate the development of neural circuits. The formation of synapses (Clarke and Barres, 2013) and the modulation of synaptic activity and plasticity by astrocytes (Araque et al., 2014) mainly take place as a result of the secretion of neuroactive substances now known as synaptogenic factors. Pfrieger and Barres (1997) were the first to demonstrate the key role of astrocyte-derived soluble molecules in synapse formation, and many subsequent studies by the same group and others have identified the nature of these synaptogenic factors, with the thrombospondins 1- 5, hevin, and glypicans seeming to be crucial substances for the formation of excitatory synapses (Ullian et al., 2001; Christopherson et al., 2005; Kucukdereli et al., 2011; Allen et al., 2012). Overall, these findings are seminally important as they have changed our “neurocentric” view of the astrocytic-dependent processes involved in the formation and maturation of functional synapses. However, we are still only beginning to understand the role of astrocyte-derived synaptogenic molecules in terms of cell biology, and our knowledge of the molecular and cell mechanisms regulating the active release of synaptogenic factors is very limited (Petrelli and Bezzi, 2016). Many of them have only been studied in cultured cells, and the cellular and molecular pathways governing their secretion are still unknown, including whether they are calcium-dependent or not. Interestingly, astrocytes also release many other factors, such as substances regulating metabolism, the energy supply (including cerebral blood flow) and inflammation, and substances regulating synaptic transmission, including neurotransmitters and neuromodulators (i.e., gliotransmitters) (Bezzi and Volterra, 2001; Petrelli and Bezzi, 2016). The cell mechanisms governing the release of gliotransmitters and their effects on synaptic activities have been extensively studied, and there is a large amount of experimental evidence indicating that gliotransmitters play an active role in synaptic physiology (Araque et al., 2014). For example, the tumor necrosis factor alpha (TNFα) released by astrocytes (and possibly also by microglial cells) is required for synaptic scaling (Stellwagen and Malenka, 2006), and can control glutamatergic gliotransmission under both physiological (Santello et al., 2011) and pathological conditions (Bezzi et al., 2001; Habbas et al., 2015). A recent study has shown that pathological concentrations of pro-inflammatory TNFα signal through astrocytes to alter synaptic transmission and impair cognition in a mouse model of multiple sclerosis (Habbas et al., 2015). As inflammation of the CNS is a common feature of nearly all neurological disorders and insults (including ASDs), and cognitive impairments are also common to many neuroinflammatory neurological conditions (including ASDs), it is likely that the activation of glial cells and pathological concentrations of cytokines (notably TNFα) can contribute to the cognitive and behavioral impairments characterizing ASDs.

Microglial cells are resident macrophages of the brain that form the innate defensive system (Hanisch and Kettenmann, 2007; Kettenmann, 2011) and, by acting as sentinels, can detect the first signs of pathogenic invasion or tissue damage. After their conversion from a resting (or surveillance) cell type to an activated form, they remove damaged synapses and their protective or detrimental functions have been extensively studied in various brain pathologies (Kettenmann et al., 2013). During the resting/surveillance phase, microglial processes constantly extend and retract to check the local environment (Nimmerjahn et al., 2005), which includes peri-synaptic astrocytes, pre-synaptic boutons, and post-synaptic spines (Tremblay et al., 2010). Many of their functions are still unclear and there has been much debate about whether they are beneficial or detrimental. Like astrocytes, microglial cells have heterogeneous functions within the brain (Carson et al., 2007; Bailey-Bucktrout et al., 2008; Bulloch et al., 2008; Gowing et al., 2008), and release a wide variety of molecules (e.g., brain-derived neurotrophic factor, BDNF) that can interact with synapses and play important physiological roles in learning and memory (Parkhurst et al., 2013).

**FIGURE 1:**
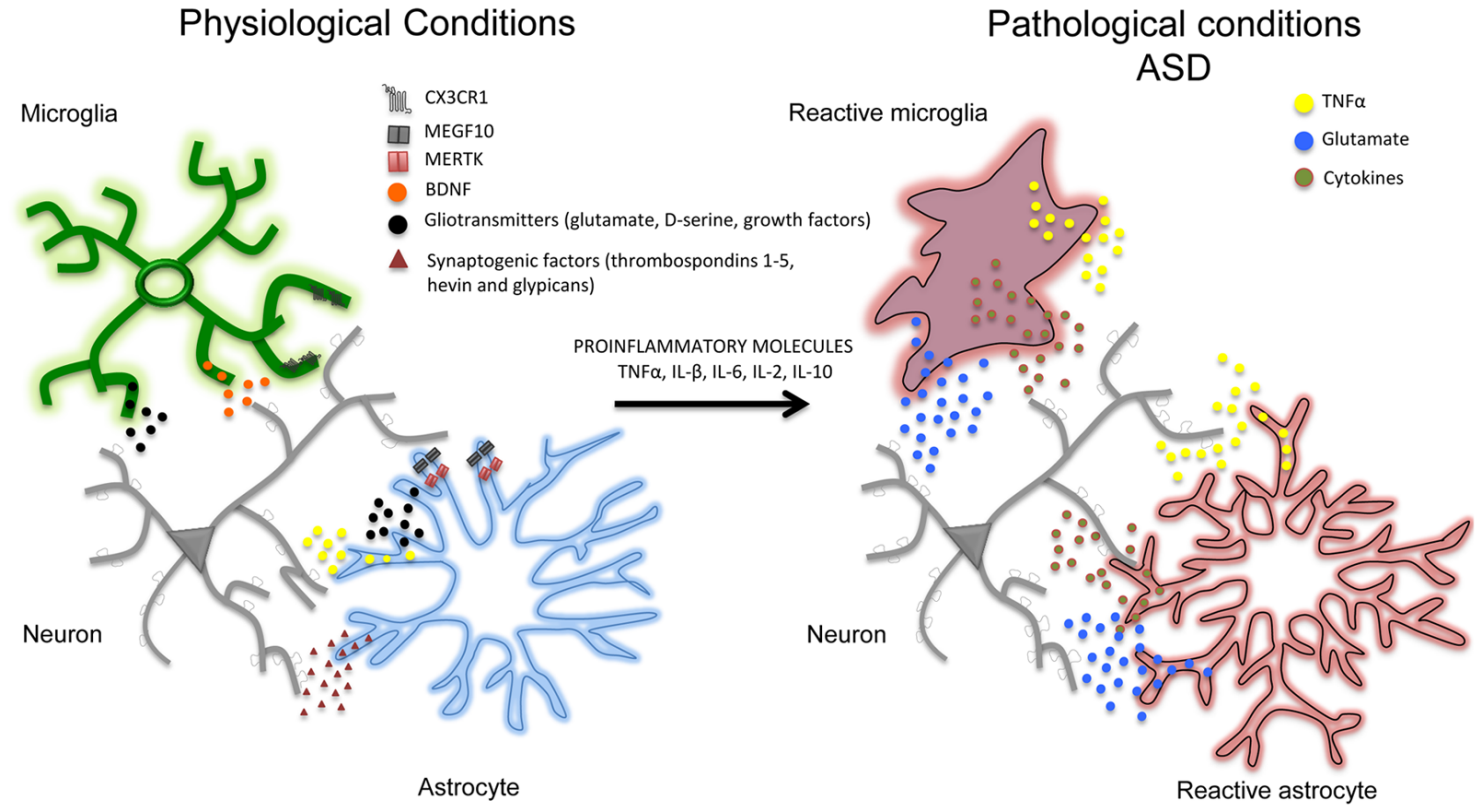
**In physiological conditions and during postnatal development, neurons (gray) and glial cells (notably astrocytes –blue- and microglia –green-) are closely associated with each other.** Astrocytes and microglia release a number of neuroactive substances such as gliotransmitters together with growth factors (such as TNFα, BDNF, glutamate, D-serine; black dots) and synaptogenic factors (such as thrombospondins 1-5, hevin, and glypicans; red dots) that can promote the formation and maturation of synapses. Glial cells can also contribute to synapse remodeling and pruning through CX3CR1, multiple EGF-like domains 10 (MEGF10), c-mer proto-oncogene tyrosine kinase (MERTK) phagocytic pathways. In pathological conditions (i.e., in autistic brains) pro-inflammatory factors (including pro-inflammatory cytokines TNFα, IL-β, IL-6, IL-2, IL-10) may lead to a chronic neuroinflammation in which astrocytes and microglia become reactive (red), release pro-inflammatory mediators (such as TNFα, glutamate and pro-inflammatory cytokines) and may exacerbate the initial inflammatory condition. Although the specific contribution of aberrant glial cells (i.e., reactive gliosis) to the pathophysiology of autism has still to be determined, studies on animal models support a direct association between neuroinflammation and autism, suggesting that reactive gliosis may have a crucial role in many autism phenotypes including the structural and functional alterations of brain connectivity.

In addition to affecting synaptic activity, astrocytes and microglial cells seem to play an important role in removing (‘pruning’) synaptic connections (Stanfield et al., 1982; Nakamura and O’Leary, 1989), a process of structural formation and elimination that is vital for controlling and refining the connectivity of mature neuronal circuits (Steven et al., 2007; Barres, 2008). For example, in the developing brain, astrocytes can physically eliminate synapses through the multiple EGF-like domains 10 (MEGF10) and c-mer proto-oncogene tyrosine kinase (MERTK) phagocytic pathways (Chung et al., 2013) and, in line with this, mice deficient in MEGF10 and MERTK receptors fail to refine their retino-geniculate connections and retain excess functional synapses. Interestingly, the rate of synaptic pruning decreases with age and is regulated by synaptic activity. Like astrocytes, microglia cells are also important in the structural elimination of synapses in developing brain. Microglia processes are highly motile and are well positioned to interact with boutons and spines. However, synaptic pruning particularly involves phagocytic microglial processes that, during the first weeks after birth, can engulf pre- and post-synaptic elements (Paolicelli et al., 2011; Schafer et al., 2012). This early post-natal process depends of different molecules such as CX3C chemokine receptor 1 (CX3CR1), also known as the fractalkine receptor or G-protein coupled receptor 13 (GPR13), and complement receptor 3 (CX3C) (Harrison et al., 1998; Stevens et al., 2007; Zhan et al., 2014; Wu et al., 2015). The importance of the CX3CR1-mediated mechanisms by means of which microglial cells remove synapses has been confirmed by studies of CX3CR1 knockout animals, which have aberrant long-range functional connectivity (Schafer et al., 2012) as well as a deficit in hippocampal LTP and aberrant social behavior (Zhan et al., 2014). Overall, these studies show that physiological interactions of astrocytes and microglia with synapses are crucial for synapse formation and network functioning, and point out that their loss, deviation or functional perturbation might contribute to autism pathogenesis and progression (**Figure 1**).

## The Role Of Glial Cells In Autism Spectrum Disorders

The importance of glial cells in the pathophysiology of ASDs is primarily supported by a recent transcriptomic analysis of autistic brains. RNA sequencing revealed a close association between ASDs and the genes related to glial cell activation and genes belonging to immune and inflammatory categories (Voineagu et al., 2011). These transcriptional results are supported by immunohistochemistry data obtained from human post mortem brain samples showing increased reactive gliosis and glial cell proliferation (Purcell et al., 2001; Vargas et al., 2005; Fatemi et al., 2008; Morgan et al., 2012; Tetreault et al., 2012; Edmonson et al., 2014), and by a recent positron emission tomography (PET) functional imaging study showing microglial activation in multiple brain regions of young adults with ASDs (such in the cerebellum, midbrain, pons, fusiform gyri, and the anterior cingulate and orbitofrontal cortices; Suzuki et al., 2013). In line with this, high levels of various pro-inflammatory cytokines, such as interleukin (IL)-6, TNFα, and IL-1β have been reported in the post-mortem brain tissues, (Watkins et al., 2001; Vargas et al., 2005; Li et al., 2009; Wei et al., 2011) and blood of autistic subjects (Gupta et al., 1998; Jyonouchi et al., 2001).

It is now recognized that pro-inflammatory factors play an important role in the etiology of various neurological and neuropsychiatric disorders, including those such as ASDs whose pathogenetic onset occurs during early brain development (Meyer et al., 2011; Voineagu et al., 2011; Gupta et al., 2014; Estes and McAllister, 2015). The developing brain is highly vulnerable to environmental insults such as those associated with strong inflammatory reactions (Dammann and Leviton, 2004) and specific brain lesions (Hagberg et al., 2015). Under normal conditions, inflammatory processes in the developing brain are controlled by a number of homeostatic mechanisms that limit the inflammation induced by an environmental stimulus such as infection (Serhan and Savill, 2005). These surveillance mechanisms are mainly controlled by microglia and astrocytes, and are crucial in ensuring that inflammatory processes efficiently remove invading pathogens and contribute to tissue repair.

It is therefore likely that, under inflammatory conditions, any dysfunction in these mechanisms may lead to deleterious chronic inflammation, and this (together with reports of increased levels of pro-inflammatory cytokines in the post-mortem brains of subjects with ASDs) has led to the hypothesis that chronic neuroinflammation plays a role in the pathogenesis of ASDs, which is supported by the fact that chronic systemic inflammatory conditions such as those associated with autoimmune disorders or infections (together with acute immune activation) are often observed in the mothers of children with ASDs (Atladottir et al., 2009; Keil et al., 2010; Estes and McAllister, 2015). Although the specific contribution of maternal immune dysregulation to the onset of ASDs has yet to be determined in humans, animal models have shown a causal link between the activation of the maternal immune system and altered neurodevelopment. Studies of various animal models of maternal infection during pregnancy support the association between systemic inflammatory processes and ASDs phenotypes (Malkova et al., 2012; Knuesel et al., 2014; Missault et al., 2014), all of which seem to indicate that the ASD-like behavior in the offspring may be caused by altered levels of maternal cytokines, including TNFα, IL1β, IL-2, IL-6, and IL-10 (Ponzio et al., 2007; Smith et al., 2007). It is important to note that there is a specific temporal window in which cytokines confer a risk of developing ASD (i.e., the perinatal period), which suggests that the permeability of the developing blood brain barrier is crucial for the onset of ASD (Meyer et al., 2011). Pro-inflammatory cytokines in the developing brain classically lead to neuroinflammation, a condition in which microglia and astrocytes become reactive (gliosis), proliferate and, depending on the entity of the gliosis, recruit peripheral leukocytes and thus amplify the initial tissue damage (Sofroniew, 2015).

The idea that reactive gliosis may exacerbate the inflammatory conditions caused by immune activation and contribute to the pathogenesis of ASDs is intriguing, but it is still not clear what the changes in glial cell activation tell us about the molecular and cellular mechanisms underlying the disorders. One possibility is that reactive gliosis may perturb the ability of microglia and astrocytes to modulate the maturation, elimination (phagocytosis), or functioning of developing synapses because developing neural networks are highly vulnerable to insults affecting the glial pathways governing the pruning of synapses (Chung et al., 2015). For example, CX3CR1 knockout mice show impaired synaptic pruning, social behavior and functional connectivity (Zhan et al., 2014), all of which are features of ASDs. It can therefore be expected that the inflammatory processes targeting the developing brain have a long-lasting impact on brain and behavioral functions throughout life.

The use of animal models of ASDs has led to substantial advances in our understanding of the role of glial cells, which have been found to be abnormal in mouse models of Rett syndrome (RTT; Maezawa et al., 2009; Yasui et al., 2013), fragile X syndrome (Yuskaitis et al., 2010), and tuberous sclerosis (Uhlmann et al., 2002). In particular, a study of a mouse model of RTT, a devastating neurodevelopmental disorder caused by loss-of-function mutations in the X-linked MECP2 encoding methyl-CpG-binding protein 2 (MeCP2) (Chahrour and Zoghbi, 2007), has shown that MeCP2-deficient microglia cells release an abnormally high level of glutamate, causing excitotoxicity that may contribute to dendritic and synaptic abnormalities (Maezawa and Jin, 2010). MECP2 is a well-known transcription factor that is important in controlling gene expression by interpreting and regulating epigenetic markers (Chao and Zoghbi, 2012). MECP2 is expressed in many tissues but, although the disease is generally attributed to a primary neuronal dysfunction, glial MECP2 seems to play a pathophysiological role as it has been recently shown that MECP2-null astrocytes are unable to support the normal dendritic ramification of wild-type neurons growing in culture (Ballas et al., 2009), and two remarkable studies have found that the expression of wild-type MECP2 protein in the astrocytes or microglia of MECP2-null hosts dramatically improves the pathology (Lioy et al., 2011; Derecki et al., 2012).

Finally, it is interesting to note that a recent RNA-Seq transcriptome and splicing database of glia and neurons (Cahoy et al., 2008) indicates that many of the ASD candidate genes are enriched in glial cells. For example, some genes, such as Homer1 (HOMER1) (Ronesi et al., 2012), 4-aminobutyrate aminotransferase (ABAT; Barnby et al., 2005), fatty acid binding protein 7 (FABP7; Maekawa et al., 2010), and glutathione *S*-transferase 1 (GSTM1; Ming et al., 2010), are not specific for neurons but, instead, are highly enriched in astrocytes; other genes are equally present in astrocytes and neurons (such as MeCP2), and some are highly enriched in both astrocytes and microglial cells (such as dual-specificity tyrosine-(*Y*)-phosphorylation- regulated kinase 1A or DYRK1A). Mutations in the genes encoding a number of members of the IL-1 cytokine receptor family are also associated with ASDs, and glial cells are the major contributors to the response of IL-1 signaling pathways to neuroinflammation (Moynagh et al., 1993; Zhang et al., 1996; Molina-Holgado et al., 2000). For example, recent exome-sequencing studies of ASD patients have found a single-nucleotide polymorphism in the gene encoding IL-1 receptor type 2 (IL-1R2) (O’Roak et al., 2011; Sanders et al., 2012) that is highly enriched in microglial cells (Cahoy et al., 2008), and a rare ASD-associated mutation has been identified in the gene encoding IL-1 receptor accessory protein –like 1 (IL-1RAPL1; Bhat et al., 2008), which is highly enriched in astrocytes (Cahoy et al., 2008).

## Future Perspectives

Until very recently, the role of glial cells in the onset of ASDs was almost completely overlooked, and so neuropharmacological strategies for treating the symptoms were almost exclusively aimed at neuronal activity and synaptic transmission. However, as accumulating evidence supports the view that astrocytes and microglia are significantly involved in the regulation of synapse formation, function, plasticity, and elimination, the role of astrocyte-derived factors in regulating synapse formation and the role of microglia in synaptic pruning during postnatal development (a period that coincides with the onset of many ASDs) are particularly relevant. These findings may change our “neurocentric” view of the mechanisms involved in the onset and progression of ASDs because that it is unlikely that research solely concentrated on neurons will fully reveal their underlying pathophysiological mechanisms. Recent data suggest that many ASDs are at least partially due to disorders affecting glial cells or neuron-glial interactions, and future pharmacological research should consider the possibility of improving glial cell functions.

## Author Contributions

PB wrote the review together with FP and LP.

## Conflict of Interest Statement

The authors declare that the research was conducted in the absence of any commercial or financial relationships that could be construed as a potential conflict of interest.
